# Anti-Inflammatory Interleukin Levels Reflect Th1/Th2 Imbalance in Spondyloarthritis Patients with Concomitant Atopy Under Biological Therapy

**DOI:** 10.3390/jcm14093094

**Published:** 2025-04-30

**Authors:** Georgiana Strugariu, Cristina Pomirleanu, Mara Russu, Vladia Lapuste, Daniela Constantinescu, Petru Cianga, Codrina Ancuta

**Affiliations:** 1 Rheumatology and Rehabilitation Discipline, Faculty of Medicine, “Grigore T. Popa” University of Medicine and Pharmacy of Iasi, 700115 Iasi, Romania or georgiana_strugariu@yahoo.com (G.S.);; 2 2nd Rheumatology Department, Clinical Rehabilitation Hospital, 700661 Iasi, Romania; 3 Immunology Discipline, 1st Morpho-Functional Sciences Department, Faculty of Medicine, “Grigore T. Popa” University of Medicine and Pharmacy of Iasi, 700115 Iasi, Romaniapetru.cianga@umfiasi.ro (P.C.); 4 Sf. Spiridon Clinical Hospital, 700111 Iasi, Romania

**Keywords:** interleukins Th2, atopy, spondylarthritis, biological therapy

## Abstract

**Background/Objectives**: Atopy and spondyloarthritis (SpA) are immune-mediated diseases driven by distinct T-helper (Th) cell pathways—Th2 for atopy and Th1/Th17 for SpA. The coexistence of these divergent immune responses is increasingly recognized, particularly in the context of biological therapies that target pro-inflammatory cytokines. This study aimed to investigate Th2 cytokine profiles (IL-4, IL-5, IL-13) in atopic SpA patients receiving biological therapy to better understand how such treatment may influence immune regulation in this complex clinical setting. **Methods**: We conducted a prospective observational cross-sectional study on 136 SpA patients stratified by biological therapy and atopy status. Serum IL-4, IL-5, and IL-13 levels were quantified using LUMINEX immunoassays. Patients were grouped into biologically treated (BT) and Bio-Naïve (BN) cohorts and further sub-categorized by atopic phenotype (allergic rhinitis, asthma, dermatitis). Statistical comparisons of cytokine levels were made using SPSS IBM version 26 to explore associations with clinical and demographic variables. **Results**: IL-13 levels were significantly elevated in BT-atopic patients, particularly those with allergic rhinitis and atopic dermatitis, suggesting biological therapy may modulate Th2 responses. IL-5 remained elevated in allergic asthma cases despite treatment, indicating persistent eosinophilic activity. No significant correlation was found between cytokine levels and disease duration or therapy length. **Conclusions**: Biological therapy in SpA may influence Th2 cytokine expression, notably IL-13, in atopic patients. These findings underscore the importance of immune profiling in guiding personalized treatment strategies and highlight the need for further investigation into the long-term immunomodulatory effects of biologics in patients with overlapping Th1/Th2-driven diseases.

## 1. Introduction and Objectives

### Atopy and Spondyloarthritis (SpA): Intersecting Immune Pathways

Atopy and SpA are immune-mediated conditions driven by distinct CD4+ T cell responses: atopy by Th2 (IL-4, IL-5, IL-13) and SpA by Th1/Th17 (TNF-α, IFN-γ, IL-17A) pathways [1,2]. However, recent evidence shows these immune axes can coexist, particularly in chronic inflammatory rheumatic diseases such as psoriasis, Crohn’s disease, and rheumatoid arthritis [3,4,5]. Immune homeostasis depends on a tightly regulated balance among Th1, Th2, and Th17 pathways, and dysregulation may contribute to overlapping disease phenotypes and persistent inflammation [6,7,8].

SpA encompasses a group of chronic inflammatory diseases affecting the axial and peripheral skeleton, with extra-skeletal involvement in the skin, gut, and eyes [9]. Though traditionally Th1-dominant, SpA involves complex Th1/Th17, Th1/Th2, and Th2/Th17 interactions. Th17 cells, via IL-17A, contribute to chronic inflammation in joints and entheses [7,8,9,10,11]. The IL-23/IL-17 axis, part of a type 3 immune response, plays a central role, with varying IL-23 dependency across SpA subtypes. For instance, IL-17A production in PsA is IL-23-dependent, whereas in AS and axial SpA, it is IL-23-independent [8,9]. Th2 responses also influence SpA pathogenesis. In some patients, diminished Th2 activity may fail to counteract Th1 inflammation [9,10]. Conversely, intense Th1-driven inflammation may induce Th2 cytokines such as IL-4, IL-5 and IL-13 [7,8,9], primarily involved in antibody production and allergic responses [2,3], also cut in mitigating Th1-mediated inflammation, further destabilizing immune regulation. Moreover, Th2 cytokines can also suppress pro-inflammatory mediators, including IL-6, TNF-α, IL-17A, and other inflammatory factors such as macrophage inflammatory protein-3, prostaglandins, reactive oxygen species, metalloproteinase or granulocytes-macrophage colony-stimulating factor [11]. In psoriasis, IL-4/IL-13 reduces IL-12, attenuating the IL-12–IFN-γ–Th1 axis. Their role in arthritis remains unclear, with evidence suggesting time-dependent expression and variable effects on joint inflammation [11,12]. The Th2/Th17 axis, though less studied, may also regulate SpA [7,13,14]. IL-4/IL-13 suppresses IL-17A and IL-23, while IL-17A may inhibit Th2 responses by inducing IL-13 decoy receptors [7,11,15]. This interplay could explain why IL-17/IL-23 blockade sometimes triggers atopic dermatitis in psoriasis patients [7,11]. However, the role of IL-13 in SpA remains poorly understood [16].

Regulatory T cells (Tregs) are vital for controlling immune responses and maintaining tolerance [17,18]. In SpA, reduced peripheral CD4+ Treg levels and increased synovial Tregs suggest recruitment to inflamed sites [18]. Zhao et al. reported that untreated AS patients at disease onset exhibited reduced circulating CD4+ CD25+ CD127low/- Treg levels but increased intra-articular Tregs, leading to impaired suppression of IFN-γ and IL-17 [17,18,19,20]. However, these may be insufficient or dysfunctional, failing to suppress Th1/Th17 activity [18,20]. Pro-inflammatory cytokines like IL-23 can impair Treg function, further fueling inflammation and tissue damage. Despite their presence, Tregs in SpA may be insufficient or functionally impaired, unable to effectively regulate Th1 and Th17 responses, thereby contributing to ongoing inflammation and joint damage [18,19,20]. This imbalance between Th1–Treg and Th17–Treg axes is further amplified by pro-inflammatory cytokines such as TNF-α, IL-17, and IL-23. Notably, IL-23 promotes Th17 differentiation while suppressing Treg function, perpetuating synovitis, enthesitis, and progressive joint destruction that may lead to ankylosis [18,20]. In the absence of effective Treg-mediated regulation, chronic inflammation continues to drive disease progression in SpA [17,18,19,20].

In atopic diseases, an imbalance between regulatory T cells (Tregs) and Th2 cells can significantly impact immune regulation [21,22]. While Tregs are essential for suppressing Th2-mediated inflammation and maintaining immune homeostasis, Th2 cytokines—particularly IL-4—also influence Treg differentiation [11,21,22]. IL-4 supports the development of Th2-associated Tregs, which help modulate Th2-driven responses. This relationship is bidirectional rather than purely antagonistic: IL-4 can shape Treg function, and Tregs, in turn, regulate Th2 cell activity [4,11]. However, in chronic atopic conditions, sustained IL-4 secretion may impair Treg function, weakening their regulatory capacity and disrupting immune balance [7,21,22]. This dysfunction can lead to unchecked Th2 activity, resulting in excessive IgE production, eosinophil recruitment, and persistent inflammation—hallmarks of diseases such as allergic asthma. Thus, the delicate Treg–Th2 balance plays a critical role in controlling allergic inflammation, and its disruption contributes to disease chronicity and severity [7,11,21,22].

Given the complexity of immune regulation, a new paradigm recognizes the interplay between Th1, Th2, and Th17 pathways in chronic relapsing inflammatory diseases (such as spondyloarthropathies, inflammatory bowel disease, psoriasis), which often coexist with Th-polarized conditions like atopy. This immune crosstalk has important therapeutic implications, especially in the era of biologics. Agents targeting TNF, IL-17, IL-23, and IL-12/23 are widely used in SpA and related chronic inflammatory conditions, while IL-4, IL-5, and IL-13 inhibitors are effective in atopic diseases [7,8,9,23]. However, the long-term impact of cytokine blockade on immune balance remains unclear, highlighting the need to better understand how to restore and maintain Th axis equilibrium in patients receiving biological therapy.

Approximately 24–25% of SpA patients present with coexisting atopic disorders, reflecting an underlying imbalance in the Th1/Th2/Th17 pathways [3,4,5]. In our cohort of 200 biologically treated SpA patients, we previously reported a 24% prevalence of atopy, which was associated with differences in treatment response [24]. These findings underscore the importance of further investigating how atopy modulates immune function and influences the outcomes of biological therapy.

We aimed to investigate Th2 cytokine profiles in atopic SpA patients receiving biological therapy to explore potential immune differences between biotreated and non-bitreated patients. Understanding how atopy influences immune responses, especially in those patients with biologics for their concomitant SpA, may help optimize and personalize treatment strategies.

## 2. Materials and Methods

### 2.1. Patients and Controls

We conducted a cross-sectional study on a cohort of 136 patients with spondyloarthritis, including both non-radiographic and radiographic axial SpA (classified per ASAS 2009 criteria) and psoriatic arthritis (classified per CASPAR 2006 criteria), recruited from the Rheumatology 2 Department, Clinical Rehabilitation Hospital, Iași, Romania.

**Figure 1:**
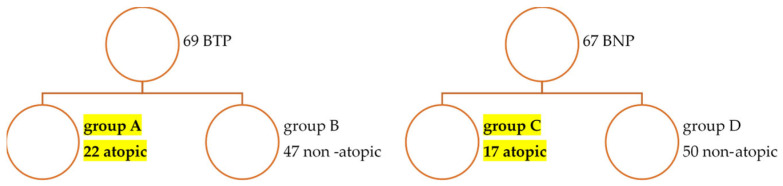
Study Group Stratification: Biologically Treated (BT) vs. Bio-Naïve (BN) SpA Patients.

Patients were stratified into two main groups based on exposure to biological therapy: Biologically Treated Patients (BT; n = 69) who had received TNF or non-TNF biological disease-modifying anti-rheumatic drugs (bDMARDs) and Bio-Naïve Patients (BN; n = 67), who had never received biological treatment. All BT patients were treated according to national guidelines and recorded in the Romanian Registry of Rheumatic Diseases (RRBR). Further stratification was performed based on confirmed atopy status (defined by diagnosis of allergic rhinitis, asthma, or atopic dermatitis by a specialist), resulting in four subgroups: Group A (BT + Atopy; n = 22), Group B (BT + Non-Atopy; n = 47), Group C (BN + Atopy; n = 17), and Group D (BN + Non-Atopy; n = 50) (Figure 1).

Each atopic patient was matched with a non-atopic counterpart based on sex, age, and disease duration. Treatment comparability was assessed across groups, considering key demographic and clinical factors to evaluate differences in treatment response and cytokine profiles.

The group of 69 BT patients was selected from a cohort of 200 patients with spondyloarthritis, previously investigated in a study on atopic disorders as comorbidities associated with SpA based on their willingness to participate in this analysis.

Our study population comprised individuals primarily undergoing rheumatologic treatment for SpA who also had a history of mild or intermittent atopic manifestations. Patients experiencing acute exacerbations of atopic disease or those with severe or difficult-to-treat atopic conditions who would qualify for Th2-targeted biological therapies were not included in this study.

### 2.2. Study Design

Clinical and demographic data were collected using a standardized protocol and included age, sex, SpA subtype, age of disease onset, disease duration, atopy subtype and onset, and treatment history. Clinical activity scores such as BASDAI (Bath Ankylosing Spondylitis Disease Activity Index), ASDAS (Ankylosing Spondylitis Disease Activity Score), DAPSA (Disease Activity in Psoriatic Arthritis), and CRP levels were recorded at the time of evaluation.

All patients included in our cross-sectional clinical study were managed in accordance with validated therapeutic protocols for spondyloarthritis [25,26]. In line with our objective to evaluate serum levels of Th2 cytokines, it is important to highlight that none of the patients in the biologic-treated (BT) group were receiving systemic immunosuppressive therapies, and glucocorticoid use was infrequent. Similarly, in the biologic-naïve (BN) group, no systemic glucocorticoids or potent immunosuppressive agents were administered, consistent with established SpA treatment guidelines. A notable exception was two patients with psoriatic arthritis and polyarticular involvement, who were treated with medium-dose methotrexate.

### 2.3. Serum Cytokine Measurement

Serum levels of Th2 cytokines (IL-4, IL-5, IL-13) were measured using LUMINEX multiplex immunoassay technology on 5 mL blood samples processed at the Immunology Department, University of Medicine and Pharmacy “Grigore T. Popa”, Iași, Romania.

Specifically, we employed the commercial LUMINEX Discovery Assay, Human Premixed Multi-Analyte Kit LXSAHM-11 (R&D Systems, Minneapolis, MN, USA) and a Gen-Probe LUMINEX 100/200 xMAP platform (Austin, TX, USA).

All samples were processed strictly following the manufacturer’s instructions. Standard curves for each cytokine (6-point curves) were automatically generated based on serial dilutions of the provided standard solutions. The LUMINEX x PONENT, version 4.3u1 (LUMINEX Corporation, Austin, TX, USA) software interpolated the mean fluorescence intensity (MFI) values of the samples onto the standard curve to calculate the corresponding analyte concentrations.

### 2.4. Group Matching Considerations

Although a formal propensity score matching (PSM) methodology was not applied due to the observational and cross-sectional nature of the study, we sought to minimize confounding by stratifying patients based on clinically relevant variables such as age, sex, disease duration, CRP levels, and disease subtype. This approach aimed to ensure a balanced comparison between biologically treated and Bio-Naïve SpA patients with or without atopic conditions while remaining within the limits of the available dataset. Future prospective studies will be needed to apply more rigorous matching strategies.

### 2.5. Statistical Analysis

Various statistical methods were employed to analyze data: descriptive statistics (mean, median, standard deviation, skewness) summarized key data characteristics, while graphical representations illustrated group distributions. To assess differences between two independent groups, the assumptions for applying the independent samples *t*-test were first evaluated. The normality of the dependent variable within each group was tested using the Shapiro–Wilk test. If normality was confirmed (*p* > 0.05), the independent samples *t*-test was applied, with Levene’s test used to assess the homogeneity of variances. The appropriate test result (assuming equal or unequal variances) was interpreted accordingly. In cases where the Shapiro–Wilk test indicated a significant deviation from normality (*p* < 0.05), the non-parametric Mann–Whitney U test was employed, as it does not assume a normal distribution and is suitable for comparing medians between two independent groups.

Comparative analyses included independent paired *t*-tests for the assessment of Th2 cytokine (IL-4, IL-5, IL-13) variations across atopic subgroups within each treatment group. Correlation analysis, using Pearson’s Mann-Whitney U test for small groups, examined associations between cytokine levels and atopy subtypes. Additional analyses explored relationships between Th2 cytokines and atopy manifestations across SpA subgroups.

Chi-square tests assessed associations between categorical variables, while moderator analysis employed regression models with interaction terms to evaluate variable influence. Statistical significance was determined using α thresholds of 0.01 (highly stringent), 0.05 (standard), and 0.10 (lenient), with results considered significant for *p* < α.

All analyses were conducted using SPSS v.26.

The study was approved by the Ethics Committee of the Clinical Rehabilitation Hospital and the University of Medicine and Pharmacy Grigore T. Popa, Iași, Romania. Written informed consent was obtained from all participants.

## 3. Results

### 3.1. Characteristics of BT and BN SpA Patients in the Main SpA Cohort

#### 3.1.1. Epidemiologic Data for Main SpA Cohort Comparative Between Biotreated (BT) and Bio-Naïve (BN) (Table 1)

- Demographics

The BN-SpA group (67 patients) is 55% male, while the BT-SpA group has a higher male proportion (74%). The mean age is comparable (~50 years). Axial SpA predominates (>60%) in both groups, with a similar age of onset. Disease duration is longer in BT-SpA (18.01 ± 9.98 years) than in BN-SpA (11.83 ± 8.72 years) though not statistically significant

- Disease-Related Characteristics

Disease activity levels were high in both groups, with significant differences (*p* < 0.05) at cytokine blood tests. Elevated BASDAI (>4), ASDAS-CRP (>2.1), and CRP (>2 mg%) were more frequent in BT-SpA. PsA activity (DAPSA > 16) affected over 40% of both groups (8/16 BT-SpA, 12/26 BN-SpA). However, mean CRP levels differed significantly (*p* < 0.01), correlating with biological treatment.

- Therapeutic Characteristics

BT-SpA patients mainly received anti-TNF agents (64 cases), with adalimumab (62%) and etanercept (23%) most used. IL-17 inhibitors (iIL-17) were given to 21 patients, with 16 using them as second or third-line therapy. Biological exposure correlated significantly with disease onset (*p* < 0.001), diagnosis (*p* < 0.001), and biological switches (*p* < 0.001). The number of biologics needed for disease control correlated with years since diagnosis (*p* < 0.05).

#### 3.1.2. Characteristics of Atopic SpA Groups (Table 2)

In our cohort of 136 patients, atopy was present in 28.7% (39 cases), affecting 25% of BN-SpA and 39% of BT-SpA patients. Atopic rhinitis (AR) was the most common (61.5%), followed by allergic asthma (AA, 33.3%) and atopic dermatitis (AD, 3.9%). AR was more prevalent in BT-SpA (73%) than BN-SpA (47%), while AA was more frequent in BN-SpA (47%) than BT-SpA (22.7%). AD was rare in BN-SpA (2 cases).

The atopic SpA cohort had an equal sex distribution (20 males, 19 females), with mean ages of 47.55 ± 8.88 years (BT-SpA) and 51.47 ± 12.83 years (BN-SpA). Axial SpA was more common than PsA (77% in BT-SpA, 65% in BN-SpA). Atopy developed after age 30 in 69.2% of cases, predominantly in BT-SpA (77.3%).

BN-SpA patients had significantly higher disease activity, with elevated CRP and DAPSA scores, while BASDAI and ASDAS scores for axial SpA showed no significant differences between groups.

In the BT group, biological therapy—primarily TNF inhibitors (n = 17) and secukinumab (n = 5)—was initiated according to established clinical protocols, with continuation of conventional DMARDs (cDMARDs) not being mandatory. At the time of cytokine assessment, half of the patients (n = 11) were on their first biologic, while the remainder had received between two and five prior biological agents. Based on the Assessment of SpondyloArthritis international Society (ASAS) criteria [27], this subgroup was considered difficult to treat due to their complex therapeutic history and prior biological exposure.

#### 3.1.3. Th2 Cytokines Panel in Atopic SpA Patients

**Figure 2:**
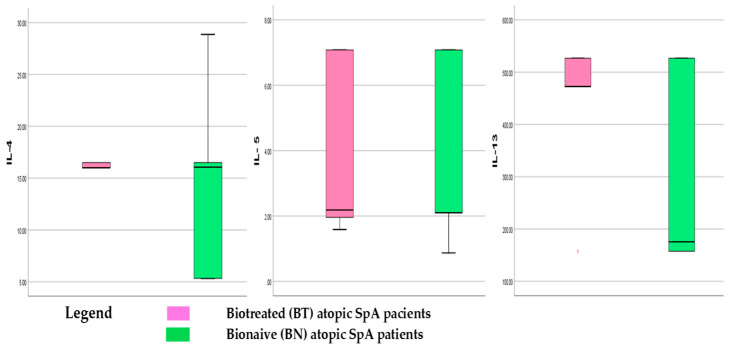
Th2-Ck in atopic SpA comparative between BN (green) and BT (pink) groups.

IL-4, IL-5, and IL-13 levels were measured in biologically treated (BT) and Bio-Naïve (BN) atopic SpA patients (Table 3), with results expressed in pg/mL based on the LLOQ from the standard curve. Trends in Th2 cytokine dynamics across groups are depicted as box-plot graphics in Figure 2.

In the BT group, IL-4 levels showed an increasing trend, while IL-5 remained unchanged. IL-13 exhibited a 1.6-fold increase or +63.8% (*p* = 0.0006), reaching a median value of 470 ± 79.55 pg/mL, indicating a rising tendency under biological treatment. Il-4 and IL-13 show a more stable value across the BT group, meaning that biologics stabilize the rising levels.

The BN group displayed high variability in pro-allergenic interleukin levels (Figure 2). Significant differences were observed between BN and BT groups for IL-4 and IL-13, but not IL-5. Median IL-5 levels (~2 pg/mL) were comparable in both groups. IL-4 medians were similar, though most BN patients fell below the 50th percentile. IL-13 levels were distinctly lower in BN patients (median < 200 pg/mL), yet over 50% exceeded this threshold, reaching up to 500 pg/mL in some cases.

### 3.2. Th2 Cytokines in Different Atopy Subtypes Associated with SpA in Our Study

**Figure 3:**
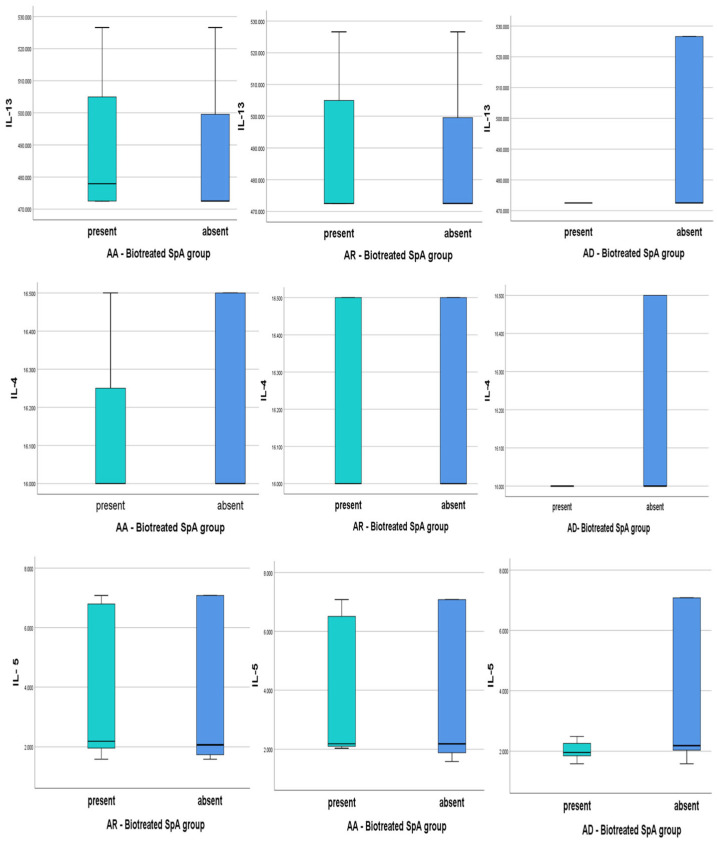
Th2 cytokines (IL-4, IL-5, IL-13) in biologically treated SPA patients with AA, AR, AD (AA—allergic asthma, AR—atopic rhinitis, AD—atopic dermatitis).

**Figure 4:**
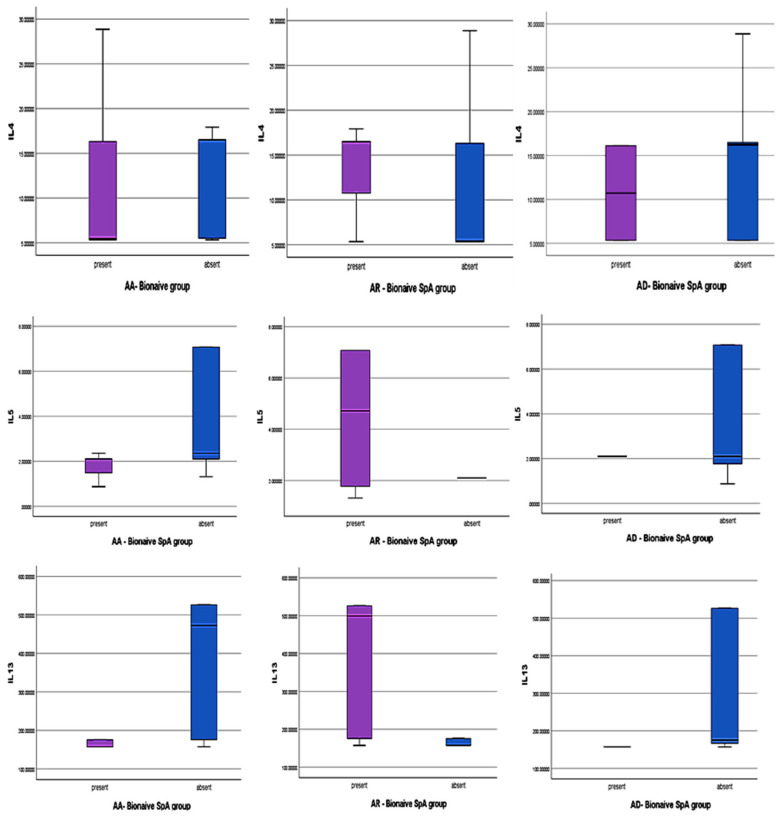
Th2 cytokines (IL-4, IL-5, IL-13) in biologically naïve SPA patients with AA, AR, AD. Legend: purple-presence of specific atopic disease and blue—for the rest of atopic disease.

Descriptive analysis of our data (min, median, max and interquartile range) is plotted in all subtypes of atopy (Figure 3 and Figure 4) in both SPA groups.

- Biologically Treated Atopic SpA and Th2 cytokines levels

*AA BT-SpA*: IL-13 levels exceed a median value of 480 pg/mL in over 50% of cases, higher than in non-AA patients. IL-4 levels remain similar across groups, suggesting no correlation with AA. IL-5 is notably elevated in AA patients, with a broader range, aligning with its role in eosinophilic inflammation. Persistent IL-5 elevation despite biological therapy (targeting IL-17/TNF) suggests residual Th2-driven inflammation, potentially benefiting from IL-5 inhibitors (e.g., mepolizumab).

*AR BT-SpA*: IL-4 and IL-13 levels are comparable to other atopic forms, with overlapping interquartile ranges, indicating AR does not influence these cytokines under biological treatment. IL-5 follows a pattern similar to AA, with a rising median and wide variability, the majority above 4 pg/mL, suggesting potential IL-5 dysregulation.

*AD BT-SpA*: no variability in IL-4 or IL-13, with consistently low levels, which may reflect immune suppression or tight regulation due to biological treatment. IL-5 is lower with a tighter distribution, indicating immune suppression or regulation under biological treatment. Non-AD patients exhibit higher IL-5 levels, suggesting a link between AD absence and Th2-driven inflammation.

No statistically significant differences were observed in IL-4, IL-5, or IL-13 levels across AR, AA, and AD *(p* > 0.05) in the BT-atopic SpA group.

- Bio-Naïve atopic SpA and Th2 cytokines levels

*AA BN-SpA*: IL-4 levels appear lower compared to other atopic patients, with greater variability in non-AA patients, suggesting IL-4 dysregulation in AA-present individuals. IL-5 levels remain low and stable across groups, while IL-13 levels are lower and exhibit minimal variability, indicating a more controlled IL-13 response.

*AR BN- SpA*: These patients display an opposing Th2 cytokine profile compared to AA-present or non-AR individuals, with significantly higher IL-5 and IL-13 levels and greater variability. IL-13 levels are particularly elevated in AR-present patients, with a median difference of ~35.6 pg/mL, highlighting its potential role in AR-associated Th2-driven inflammation. IL-4 levels are also higher in AR-present patients but exhibit lower variability, suggesting a more stable response, whereas IL-4 variability is greater in AR-absent patients, possibly reflecting alternative immune regulatory mechanisms.

*AD BN-SpA*: IL-5 and IL-13 levels are comparable between AD-present and AD-absent groups, with limited variability. However, IL-4 median levels are lower in AD-present patients, with a relatively small interquartile range (IQR), indicating stable IL-4 expression. Short whiskers further suggest minimal variability in IL-4 levels.

### 3.3. Quantitative Analysis of Mean LLOQ Levels of Th2 Cytokines in BT and BN Atopic SPA

We quantitatively analyzed Th2 cytokine levels and compared biologically treated and Bio-Naïve SpA patients across different atopic subtypes. A *t*-test was performed to assess the statistical significance of observed differences.

#### 3.3.1. The Results for AR Patients Associated with SPA in Our Groups

The results of the quantitative analysis for AR patients with SpA in our groups are represented below in Table 4.

Our results of *t*-test follow-up relief: IL-4 and IL-5 do not show statistically significant differences between the two groups (*t*-test = 0.1539, *p* = 0.142 and *t*-test = −0.875, *p* = 0.394, respectively). However, IL-13 shows a marginally non-significant difference (*t*-test = 1.860, *p* = 0.080), with a large mean difference of 101.32, but it is still above the 0.05 significance threshold.

The analysis of the mean value of Th2-IL in SpA patients with AR associated showed that the biological treatment seems to influence the mean serum levels for IL-4 and IL-13, but the *t*-test showed that only for IL-13 is weak statistical significance for a *p* < 0.1 (10%).

#### 3.3.2. The Results for AA Patients Associated with SPA in Our Groups

In Table 5, we listed the results that we obtained in AA cases comparative between biotreated and Bio-Naïve SpA patients.

#### 3.3.3. The Results for AD Patients Associated with SPA in Our Groups

The quantitative analysis for this patient subgroup is shown in Table 6. In AD patients, the mean serum levels of IL-4 and IL-13 have a rising tendency, over two-fold in the BT group, and a much more limited tendency in IL-5. It is obvious that biological treatment influences the serum levels of Th2-IL in AD SPA patients.

Because the group for AD patients consists of a very small number of patients, *t*-test analysis was not performed. However, our data showed that in the BT group, the mean levels of IL-13 rose significantly, with a difference of +322.72 pg/mL. The same dynamics we registered for IL-4 with a rising of mean levels more than 2 times.

**Figure 5:**
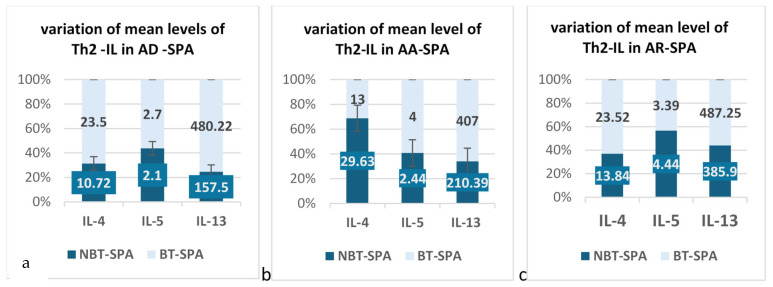
LLOQ-mean levels of Th2 cytokines in BT vs. BN SPA patients with AA, AR, AD; (**a**) variation of serum mean levels of Th2 -IL in patients with SPA and concomitant AA; (**b**) variations of serum mean levels of Th2-IL in patients with SPA and concomitant AR; (**c**) variation of serum mean levels of TH2-IL in patients with SPA and concomitant AD.

The graphical representation of changes in the mean serum levels of IL-4, IL-5, and IL-13 was analyzed in AD, AA, and AR among biotreated (BT) versus Bio-Naïve (BN) SPA patients (Figure 5a–c).

## 4. Discussion

This study highlights distinct Th2 cytokine dynamics (IL-4, IL-5, IL-13) in atopic SpA patients, particularly in those undergoing biological therapy. Our findings indicate that biologics may influence Th2 immune responses, especially IL-13, which showed significantly elevated levels in biotreated patients, particularly those with allergic rhinitis (AR) and atopic dermatitis (AD). This suggests that biological treatment, although primarily targeting Th1/Th17 pathways, may unmask or modulate Th2 responses in predisposed individuals.

Although our results showed mean cytokine levels increased in biotreated patients, especially IL-13, Pearson correlation analyses did not yield statistically significant associations with most clinical parameters, except for IL-13 in the BT-SpA group. This implies that IL-13 may be particularly responsive to biological intervention and could represent a sensitive marker for immune modulation in this setting.

A key observation is the predominance of late-onset atopy (>30 years) in the BT group (77.3%) compared to the BN group (<60%), reinforcing the possible link between biological therapy and altered Th2 activity. The association between elevated IL-13 and late-onset atopy (after age 30), particularly in BT-SpA patients, supports the hypothesis that cytokine shifts under biological therapy may contribute to the delayed manifestation or unmasking of atopic conditions.

Despite the elevation of cytokine levels, correlation analyses revealed no significant associations between IL-4, IL-5, or IL-13 levels and duration of biological therapy, disease onset, or years since diagnosis (*p* > 0.05), indicating that biological duration alone is not a determinant of Th2 cytokine levels. Instead, individual immune profiles and atopic predisposition may play more significant roles.

Interestingly, IL-5 levels were consistently elevated in BT patients with allergic asthma (AA), suggesting persistent eosinophilic inflammation despite biological therapy, more likely unaffected by TNF-α or IL-17 blockade. This residual Th2 activity—possibly due to the limited direct impact of TNF- or IL-17-blocking agents on Th2 pathways—raises the potential utility of IL-5-targeted therapies (e.g., mepolizumab) for specific atopic SpA subpopulations [24].

In our SpA patients with AD-associated, biotreated individuals showed marked increases in IL-4 and IL-13 (2–3 fold), with minimal change in IL-5. This selective cytokine upregulation suggests differential regulation of Th2 markers under biological influence, consistent with findings in psoriasis and other inflammatory conditions [28,29].

Our comparisons across atopic subtypes shed further light on the intricate and multifaceted behavior of cytokine responses. The mean value of IL-13 was significantly higher in AR patients (*p* = 0.033) and lower in AD patients (*p* = 0.006) in the BN group, indicating that IL-13 levels vary not only with treatment status but also with atopic phenotype. This finding emphasizes that atopic phenotype contributes to cytokine expression patterns beyond treatment effects alone. Additionally, the analysis of cytokine levels by age of atopy onset confirmed the prominence of late-onset atopy in BT patients, with cytokine elevations (especially IL-5 and IL-13) more evident in older individuals. This age-associated immune shift may be relevant in understanding how biologics modulate disease trajectories and allergic manifestations in SpA.

More interestingly, cytokine levels showed no meaningful association with how long the disease had been present or when it was diagnosed in our patients, pointing instead to treatment and atopy as stronger drivers of immune changes, supporting our hypothesis.

While this study provides novel insights into Th2 cytokine dynamics in atopic SpA patients undergoing biological therapy, several limitations must be acknowledged.

First, the sample size, particularly within the atopic subgroups such as allergic asthma and atopic dermatitis, was relatively small. The limited number of cases in these categories (e.g., only 2 AD patients in the bio-naïve group) reduced the statistical power and precluded more robust subgroup analyses, including formal testing in some instances. This constraint may have affected the generalizability of our findings and limits definitive conclusions regarding cytokine behavior across all atopic phenotypes. To validate these preliminary findings, future studies should incorporate larger patient cohorts, longer follow-up durations, and longitudinal assessments of cytokine profiles.

Second, although patients were matched by age, sex, and disease duration, residual confounding due to unmeasured variables such as environmental allergen exposure, concurrent medications (e.g., corticosteroids, antihistamines), and disease severity at baseline may have influenced cytokine levels. These factors were not systematically controlled or adjusted for in our analyses.

Third, the cross-sectional nature of cytokine measurements may not fully capture the dynamic fluctuations in immune responses over time or in relation to specific treatment phases. A longitudinal assessment with repeated cytokine measurements before and after initiation or switching of biological agents could provide more detailed insights into the temporal patterns of Th2 modulation.

Fourth, the study focused primarily on serum Th2 cytokines (IL-4, IL-5, IL-13) without parallel evaluation of Th1, Th17, or regulatory T cell markers. Given the complex interplay among immune pathways in SpA and atopic conditions, more comprehensive immune profiling would be beneficial to elucidate the broader immunologic shifts induced by biological therapy.

Lastly, this study did not incorporate functional assays or in vitro analyses to validate the biological significance of the observed cytokine elevations. Such mechanistic approaches would enhance the understanding of how biologics may modulate Th2 responses at the cellular level.

### Perspectives and Future Research

While our findings provide valuable insight into Th2 cytokine dynamics in SpA patients with atopic conditions, we recognize the limitations inherent in our cross-sectional design and the absence of formal matching strategies, such as propensity score matching, which could enhance the robustness of intergroup comparisons. Implementing a prospective case-control framework—focused exclusively on biologically treated patients and incorporating a comprehensive Th1/Th2 cytokine panel—represents an important avenue for future research. Although such a design was beyond the scope of the current study, our results serve as a foundational basis for hypothesis generation and underscore the need for longitudinal investigations that integrate immunologic, clinical, and therapeutic outcomes in this complex patient population.

## 5. Conclusions

Our results support a model in which biological therapy modifies Th2 cytokine dynamics, particularly IL-13, in atopic SpA patients. These findings underscore the complex immune crosstalk between Th1/Th17- and Th2-driven pathways in SpA with coexisting atopy. Persistent IL-5 activity in AA patients and elevated IL-13 in AR and AD suggest residual or redirected immune responses under biologics, warranting exploration of adjunctive therapies targeting Th2 axes.

Understanding these immunological shifts is crucial in guiding personalized treatment strategies for SpA patients with atopic comorbidities. Future studies with larger cohorts and mechanistic exploration are needed to validate these findings and refine therapeutic algorithms in this intersecting patient population to optimize therapeutic strategies for each individual.

## Figures and Tables

**Table 1:** Demographic, Disease-Related, and Therapeutic Characteristics of BT and BN SpA Patients.

General Characteristics	Bio-Naïve SpA (BN, Control Group) (n = 67)	Bio-Experimented SpA (BT, n = 69)	*p*
Gender distribution *n (%) chi-square* Male Female	37 (55) (43.35) [0.93] 30 (45) (23.65) [1.71]	51 (74) (44.65) [0.90] 18 (26) (24.35) [1.66]	0.0226 *
Mean age (years ± *SD*)	50.3 ± 11.34	49.61 ± 10.64	*p* > 0.05
SPA subtype n (%) chi-square axSpA APS PsA	41 (61) (46.31) [0.61] 26 (39) (20.69) [1.36]	53 (77) (47.69) [0.59] 16 (23) (21.31) [1.32]	0.0487
Atopy present n (%) chi-square AR AA AD	17 (25) 8 (47) (9.19) [0.15] 8 (47) (4.98) [1.83] 2 (11.7) (3.83) [0.87]	22 (39) 16 (72.7) (14.81) [0.10] 5 (22.7) (8.02) [1.14] 8 (36.3) (6.17) [0.54]	0.9834
Years since onset (medium years ± DS)	11.83 ± 8.72	18.01 ± 9.98	*p* > 0.05
Years of diagnosis (medium years ± DS)	7.43 ± 6.66	13.86 ± 9.57	*p* > 0.05
BASDAI ≥ 4 n (%) chi-square	20 (48.7) (13.96) [2.62]	12 (22.6) (18.04) [2.02]	0.007996 *
ASDAS ≥ 2.1 n (%) chi-square	24 (58.5) (18.32) [1.76]	18 (33.9) (23.68) [1.36]	0.017475 *
DAPSA ≥ 16 n (%) chi-square	12 (46) (11.82) [0.00]	8 (44.4) (8.18) [0.00]	0.910853
CRP ≥ 2 mg% n (%) chi-square	18 (26.8) (12.81) [2.10]	8 (11.6) (13.19) [2.04]	0.023557 *

* *p* < 0.05.

**Table 2:** Clinical and Demographic Characteristics of Atopic SpA Groups.

Parameter	BT Atopic SPA (n = 22)	BN Atopic SPA (n = 17)
Age (mean ± SD)	47.55 ± 8.88	51.47 ± 12.83
Gender—Male n (%)	16 (72.7%)	4 (23.5%)
axSpA, n (%)	17 (77.2%)	11 (65%)
PsA, n (%)	5 (22.8%)	6 (35%)
Years-onset (mean ± SD)	15.58± 5.81	9.85 ± 5.78
Years-diagnosis (mean ± SD)	12.37 ± 5.92	7.08 ± 5.28
Age at atopy onset 20–30 years n (%) >30 years n (%)	4 (22.7%) 17 (77.3%)	7 (41.2%) 10 (58.8%)
AR, n (%)	16 (73%)	8 (47%)
AA, n (%)	5 (22.7%)	8 (47%)
AD, n (%)	8 (36.3%)	2 (12%)
BASDAI mean ± SD	2.18 ± 2.05	2.72 ± 1.1
ASDAS mean ± SD	2.11 ± 0.97	2 ± 0.86
DAPSA mean ± SD	12.4 ± 9.70	29.6 ± 16.62
CRPmg/mL mean ± SD	0.72 ± 0.77	1.87 ± 4.03

**Table 3:** CK levels (min, max, mean, SD) in atopic patients.

CK	BT Min	BT Max	BT Mean ± SD	BN Min	BN Max	BN Mean ± SD
*IL-4*	5.33	266.25	19.68 ± 13.36	12.51	166.87	20.77 ± 35.04
*IL-5*	1.14	7.08	3.53 ± 2.39	1.44	9.83	3.36 ± 2.51
*IL-13*	12.65	526.6	450.18 ± 118.0231	160.6	526.6	288.89 ± 173.93

**Table 4:** Mean Serum Th2-IL Levels in AR: BT vs. BN SpA Patients.

Cytokines	Atopic Rhinitis Group	Number of Patients (n)	Mean	Std. Deviation	Std. Error Mean
IL-4	BT	11	23.52	17.1	5.15
	BN	8	13.84	5.23	1.84
IL-5	BT	11	3.39	2.38	0.71
	BN	8	4.44	2.84	1
IL-13	BT	11	487.25	25.27	7.61
	BN	8	385.93	180.22	63.72

**Table 5:** Mean serum values of Th2-IL levels in AA: BT vs. BN SPA Patients.

	Allergic Asthma Group	N Patients	Mean	Std. Deviation	Std. Error Mean
IL-4	BT	4	13.45	5.42	2.71
	BN	8	29.63	50.98	18.02
IL-5	BT	4	3.35	2.48	1.24
	BN	8	2.44	1.96	0.69
IL-13	BT	4	407.27	168.45	84.23
	BN	8	210.39	128.07	45.28

**Table 6:** Mean serum levels of Th2-IL for AD patients in BT vs. BN-SPA Patients.

	*Atopic Dermatitis Subgroup*	N	Mean	Std. Deviation	Std. Error Mean
IL-4	BT	7	23.45	19.50	7.37
	BN	2	10.73	7.63	5.39
IL-5	BT	7	2.76	1.92	0.72
	BN	2	2.10	0.000	0.000
IL-13	BT	7	480.22	20.44	7.72
	BN	2	157.50	0.000	0.000

## Data Availability

The data presented in this study are available on request from the corresponding author.

## Abbreviations

The following abbreviations are used in this manuscript:

SpA	Spondyloarthritis
AA	Allergic Asthma
AR	Atopic Rhinitis
AD	Atopic Dermatitis
BN	Bio-Naïve
BT	Biologically treated
INF	Interferon
IL	Interleukin
Th	T helper
TNF	Tumor Necrosis Factor
